# NEED-BASED INTERVENTION DELIVERY FOR FAMILY CAREGIVERS OF OLDER ADULTS WITH DEMENTIA

**DOI:** 10.1093/geroni/igad104.3491

**Published:** 2023-12-21

**Authors:** Jaclene Zauszniewski, Christopher Burant, Alexandra Jeanblanc, Evanne Juratovac

**Affiliations:** Case Western Reserve University, Cleveland, Ohio, United States; Case Western Reserve University, Cleveland, Ohio, United States; Case Western Reserve University, Cleveland, Ohio, United States; Case Western Reserve University, Cleveland, Ohio, United States

## Abstract

The literature on family caregivers of persons with Alzheimer’s or other dementias abounds with studies of psychoeducational or cognitive-behavioral interventions to reduce stress and promote mental health. However, the determination of the caregiver’s need for specific interventions is rarely documented or based on clinical judgment. Researchers typically assume an intervention was needed when positive outcomes emerge. In a sample of 87 family caregivers of persons with dementia, our research examined baseline cut scores on validated measures of resourcefulness and dementia knowledge, which exemplify cognitive-behavioral and psychoeducational interventions, respectively, to determine whether the caregivers needed Dementia Education or Resourcefulness Training.© We found that 66% of the caregivers showed a high need for Dementia Education and 61% showed moderate to high need for Resourcefulness Training.© More specifically, subscales comprising the Dementia Knowledge Assessment Scale revealed the highest need for education concerning the cause or course of dementia (72%) communication and behaviors accompanying the illness (79%), and associated risk factors (80%). The need for knowledge on care and symptoms was lowest (28%). Resourcefulness Scale© scores indicated a higher need for social (help-seeking) skills training (82%) than personal (self-help) skills training (36%). Interestingly, we found that 55% of caregivers showed a moderate to high need for both interventions. The findings suggest further research to investigate the delivery of Dementia Education and Resourcefulness Training© interventions based on documented caregiver need and the additive and sequential effects on stress and mental health when both interventions are provided, particularly when a need for both interventions is established.

